# A systematic review of the diagnostic accuracy of prostate specific antigen

**DOI:** 10.1186/1471-2490-9-14

**Published:** 2009-09-10

**Authors:** Philip Harvey, Amman Basuita, Deborah Endersby, Ben Curtis, Aphrodite Iacovidou, Mary Walker

**Affiliations:** 1 School of Medicine, University of Birmingham, Birmingham, UK

## Abstract

**Background:**

Prostate cancer is the fourth commonest cancer in the UK, and the incidence is rising. The reference standard for diagnosing this condition is prostate biopsy, an invasive procedure.

This study systematically reviews recent literature on tPSA accuracy. The time period was restricted to ensure that the estimates referred to contemporary tPSA tests and prostate cancer reference standards. The focus of this review was restricted to European populations as tPSA levels are known to vary by population group.

**Methods:**

Medline was searched (from 01/1998 to 01/2008) and Embase (from 01/1998 to 01/2008), which returned 3087 citations. These were assessed by 6 reviewers, who shortlisted 54 of possible relevance. 2 reviewers assessed each using the following inclusion criteria: data collection between 1998-2008; tPSA measurements for all participants; histological confirmation of the diagnosis; samples from a European population and sufficient data to calculate 2 × 2 tables. The final set of 10 included studies represented 5373 participants. Quality of the included studies was assessed in duplicate using criteria suggested by the Cochrane Collaboration. Review Manager 5.0 software was used to analyse the data, including plotting a series of summary receiver operator curve spaces (SROC).

**Results:**

tPSA sensitivities ranged from 0.78 to 1.00 and specificities from 0.06 to 0.66. Positive likelihood ratios ranged from 0.83 to 2.90 and negative likelihood ratios ranged from 0.00 to 3.75

**Conclusion:**

tPSA has a role to play as one of several indicators for prostate biopsy along with abnormal digital rectal examination and urinary symptoms. However, tPSA test has a high false positive and significant false negative rate. It is important that clinicians understand these limitations.

## Background

There were 28,886 newly diagnosed cases of prostate cancer in 2005 in England, comprising 24.1% of all cancers in men in that year. It is the fourth commonest cancer in the UK [1]. In 2005 9024 men died of prostate cancer, mostly between the ages of 80-84 [2]. The incidence of prostate cancer in European men rose from 202,100 in 2004 [3], to 301,500 in 2006 [4]. It remained the commonest cause of cancer in European men and the third commonest cause of death. The incidence is rising, partly due to our ageing population [5]. Total prostate specific antigen (tPSA) testing has risen significantly from 1999 to 2002 [6]. It is therefore important that the validity of the tPSA test be fully understood to ensure appropriate testing and referral for further investigations.

However tPSA testing was not intended as a diagnostic test, but for identifying individuals requiring further investigation [7]. NICE currently recommend the Prostate Cancer Risk Management Programme's age specific ranges for tPSA cut off levels (Table 1). NICE describe the test as moderately sensitive and specific [8]. However no evidence is provided for this, despite claiming their judgement is based on secondary research and selected primary research. Patients with a tPSA higher than the given level are recommended to undergo further investigation. However, there is great variation in clinical practice within the UK [5], with some trusts using a single cut off value of 4 ng/mL and some using the age specific ranges as shown in table 1.

**Table 1:** Watson 2002

**Age (years)**	**PSA cut-off (ng/mL)**
50-59	≥3
60-69	≥4
70+	>5

**Figure 1:**
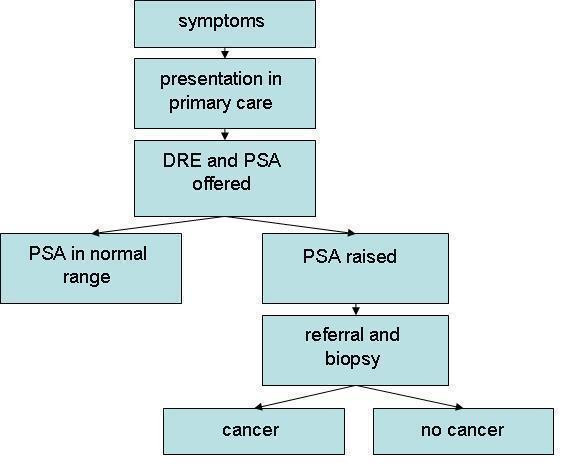
**Flow diagram depicting the diagnostic pathway for prostate cancer**.

tPSA is an inherent part of the prostate cancer diagnosis pathway. This comprises of symptoms, digital rectal examination (DRE), tPSA level and transurethral ultrasound guided biopsy (TRUS) (Figure 1). Although it is not certain what the diagnostic pathway would be like in the absence of tPSA, it seems likely that virtually all patients with suspicious clinical findings would require biopsy.

In the past few years there has been substantial debate regarding the role of tPSA as a diagnostic tool. There is a large quantity of literature available on tPSA and a simple search of the term PSA OR Prostate Specific Antigen in PubMed gives 20,469 hits. Furthermore the introduction of screening in the USA has brought discussion of whether a similar screening programme should be introduced in Europe. At the centre of this debate is the uncertainty of the diagnostic accuracy of the tPSA test. The focus of this review will be the diagnosis of prostate cancer in patients presenting with symptoms. However other forms of PSA testing such as PSA velocity, PSA density and free to total PSA ratios are not assessed. This is a reflection of clinical practice as they are of limited value and not included in the European guidance [9].

In 1999 the World Health Organisation (WHO) established a reference standard for total PSA measurement [10]. Since then, differences have decreased between the results of different assay methods [11]. Also, the Standard for the Reporting of Diagnostic Accuracy Studies (STARD) was formulated in September 2000, to improve the accuracy and completeness for reporting diagnostic accuracy studies. Its aim is to encourage a more standardised and transparent format for diagnostic test studies [12]. This review upholds these standards.

## Objectives

In this systematic review we aim to assess the sensitivity and specificity of the PSA test in the diagnosis of prostate cancer.

## Methods

### Criteria for considering studies for this review

#### Types of studies

Analytical studies assessing the accuracy of tPSA in predicting the diagnosis of prostate cancer. Only published English-language studies, carried out and published within the last 10 years (1998-2008) were considered for inclusion.

#### Participants

Men participating in studies of prostate cancer diagnosis, carried out in Europe. No age restrictions were used.

#### Index tests

Total prostate specific antigen testing. Only papers in which a full range of tPSA from <4 ng/mL to >10 ng/mL as this reflects the standard European practice.

#### Target conditions

Prostate cancer, irrespective of Gleason or TNM score.

#### Reference standards

Histological confirmation of prostate cancer either from a biopsy or resected specimen.

### Search methods for identification of studies

Electronic searches of MEDLINE and EMBASE were performed.

#### Electronic searches

The following search strategy for MEDLINE was performed via Pubmed in January 2008: ("Prostate-Specific Antigen" [Mesh] AND "Prostatic Neoplasms" [Mesh]) AND ("Diagnostic Techniques and Procedures" [Mesh] OR "Sensitivity and Specificity" [Mesh]) AND "diagnosis/broad" [Filter] AND "english and humans" [Filter] AND ("last 10 years" [PDat])

The following search strategy was used for EMBASE via OVID in January 2008:

1. exp prostate cancer/di

2. limit 1 to (human and english language and year = 1998-2008)

3. exp diagnostic techniques and procedures/

4. exp sensitivity and specificity/

5. 3 or 4

6. exp prostate specific antigen/

7. 2 and 5 and 6

### Data collection and analysis

#### Selection of studies

A list of articles meeting the inclusion criteria based on abstracts was complied. These studies and those of uncertain relevance were retrieved in full text and split into three groups based on date of publication. Two reviewers independently evaluated each group of studies for inclusion, with any discrepancies being discussed with a third reviewer until a final set of relevant studies was agreed.

#### Data extraction and management

The following data was extracted from each study:

◦ Study citation

◦ Clinical setting (clinic or screening)

◦ Participants (number, age range)

◦ Study design

◦ Characteristics of tPSA test

◦ Reference standard

◦ Study results (i.e. specificity, sensitivity, 2 × 2 table)

The data was entered into Review Manager 5.0 software.

#### Assessment of methodological quality

The methodological quality for each paper was assessed by 2 reviewers independently using the QUADAS [13] criteria [see Additional file 1].

#### Statistical analysis and data synthesis

Sensitivity, specificity, true positives (TP), false positives (FP), true negatives (TN) and false negatives (FN) were taken directly from the source papers. If this was not possible, values were calculated from the data that was provided. Positive and negative likelihood ratios, diagnostic odds ratios, and 95% confidence intervals were calculated. The data was displayed graphically on forest and SROC plots. The SROC curve was fitted using the Littenberg-Moses method.

#### Investigations of heterogeneity

Heterogeneity between studies was assessed and subgroup analyses were performed using study design as the main variable.

## Results

The search provided 3087 citations, 2580 from Medline and 492 from EMBASE. 54 were short listed and 10 were included. Studies excluded from the short list either did not fully meet the inclusion criteria or did not contain the appropriate data for our analysis. [see Additional file 2]

### Included Studies

Participant numbers of included studies varied from 59 to 3171 with a mean of 537. The total patient population was 5373. Seven studies had a prospective cohort design and three were retrospective case control studies. For detailed tables of study characteristics please see Additional file 3.

Unal 2000 and Espana 1998 included both European and non-European centres. A consensus was reached that studies should be included if all hospitals listed as research centres were European. Both studies satisfied this condition.

Espana 1998 performed two tPSA tests on each participant using the second for the main statistical analysis. Only the first test data fit the criteria for this review, therefore this data was used.

Aragona 2005 involved 16,298 participants. However only 3,171 underwent biopsy and hence only these patients were included in our analysis.

### Methodological quality of included studies

**Figure 2:**
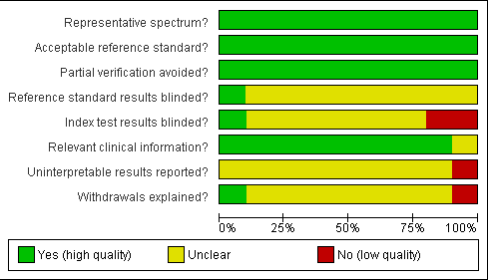
**Methodological quality graph: review authors' judgments about each methodological quality item presented as percentages across all included studies**.

Figure 2 shows the overall quality of the 10 included studies. In particular, the explanation of withdrawals and index blinding were poorly reported. It is also unclear whether uninterpretable results were reported. These are potentially important sources of bias.

**Figure 3:**
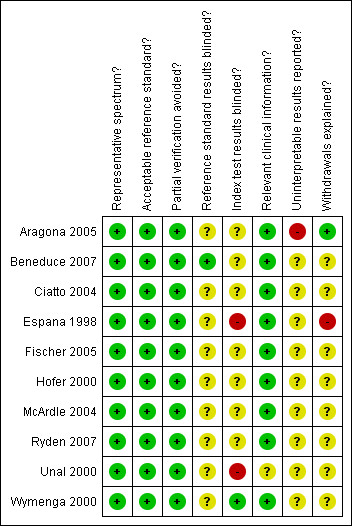
**Methodological quality summary: review authors' judgments about each methodological quality item for each included study**.

A breakdown of the methodological quality can be found in Figure 3. It can be seen that the Espana 1998 study had low quality index blinding and withdrawal explanation. The Aragona 2005 study had low quality reporting of uninterpretable results; eight of the patients in this study were lost without explanation. This is significant as this study contributed the largest proportion of our total data. The Unal 2000 study had low quality reporting of index blinding. This is perhaps less significant as this study is the smallest and blinding of the index test has a minimal effect on the PSA test and its interpretation. However the small size of this study has meant that findings, such as a false negative rate of 0, need to be interpreted cautiously.

### Statistical analysis

**Figure 4:**
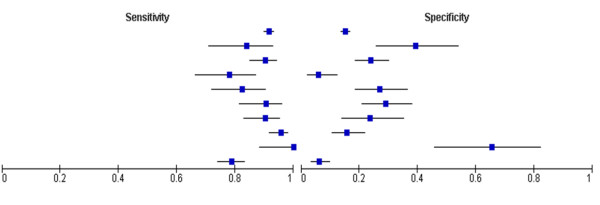
**Forest plot of sensitivity and specificity of tPSA testing**.

Table 2 displays core information collected from all included studies. This data is graphically displayed in Figure 4. Sensitivities varied from 0.78 to 1.00 (range of 0.22) and specificities ranged from 0.06 to 0.66 (range of 0.60).

**Table 2:** True positives (TP), false positives (FP), true negatives (TN), false negatives (FN), sensitivity and specificity for all studies with 95% confidence intervals.

**Study**	**Participants**	**TP**	**FP**	**FN**	**TN**	**Sensitivity [CI]**	**Specificity [CI]**
Aragona 2005	3171	1073	1695	98	305	0.92 [0.90, 0.93]	0.15 [0.14, 0.17]
Beneduce 2007	101	42	31	8	20	0.84 [0.71, 0.93]	0.39 [0.26, 0.54]
Ciatto 2004	410	167	171	18	54	0.90 [0.85, 0.94]	0.24 [0.19, 0.30]
Espana 1998	170	53	96	15	6	0.78 [0.66, 0.87]	0.06 [0.02, 0.12]
Fischer 2005	178	61	76	13	28	0.82 [0.72, 0.90]	0.27 [0.19, 0.37]
Hofer 2000	184	67	81	7	33	0.91 [0.81, 0.96]	0.29 [0.21, 0.38]
McArdle 2004	171	93	52	10	16	0.90 [0.83, 0.95]	0.24 [0.14, 0.35]
Ryden 2007	361	180	146	8	27	0.96 [0.92, 0.98]	0.16 [0.11, 0.22]
Unal 2000	59	30	10	0	19	1.00 [0.88, 1.00]	0.66 [0.46, 0.82]
Wymenga 2000	716	253	228	68	15	0.79 [0.74, 0.83]	0.06 [0.03, 0.10]

**Figure 5:**
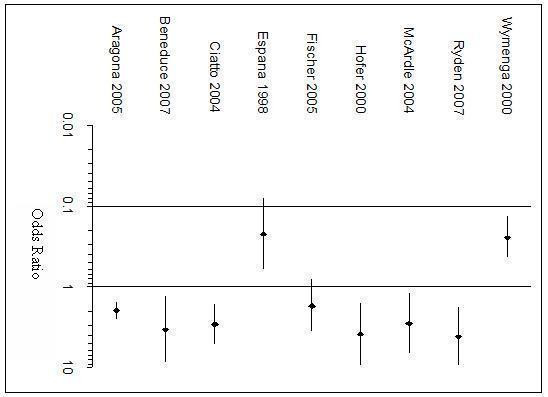
**Forest plot of Diagnostic Odds Ratios**.

Positive and negative likelihood ratios and diagnostic odds ratios (DORs) are displayed in Table 3. DORs are displayed in a forest plot in Figure 5. Only 9 of the 10 studies were plotted as the confidence intervals (CIs) of the odds ratio for the Unal 2000 study could not be calculated.

**Table 3:** likelihood ratios and diagnostic odds ratios with 95% confidence intervals

**Study (year)**	**Positive diagnostic likelihood ratio**	**Negative diagnostic likelihood ratio**	**Diagnostic odds ratio with 95% confidence intervals**
Aragona 2005	1.081	0.549	1.970 [1.550, 2.505]
Beneduce 2007	1.382	0.408	3.387 [1.320, 8.690]
Ciatto 2004	1.188	0.405	2.930 [1.650, 5.204]
Espana 1998	0.828	3.750	0.221 [0.081, 0.603]
Fischer 2005	1.128	0.653	1.729 [0.826, 3.620]
Hofer 2000	1.274	0.327	3.899 [1.622, 9.378]
McArdle 2004	1.181	0.413	2.862 [1.211, 6.762]
Ryden 2007	1.135	0.273	4.161 [1.835, 9.435]
Unal 2000	2.900	0.000	Infinite
Wymenga 2000	0.840	3.432	0.245 [0.136, 0.440]

All studies have a DOR above 1 except Espana 1998 and Wymenga 2000. Fischer 2005 has an odds ratio of 1.729 but the CI crosses 1. The remaining six studies all have their odds ratios above 1 and CIs that do not include 1, implying that the positive association of tPSA with prostate cancer is not accounted for by chance alone in these studies. Aragona 2005 has a very narrow CI compared to the other studies, which may be attributed to its large size.

Positive likelihood ratios (PLR) were above 1 for all studies except Espana 1998 and Wymenga 2000, indicating that a raised tPSA is associated with prostate cancer. However all PLRs are below 10, the threshold generally accepted for a useful test. The same eight studies have negative likelihood ratios (NLR) less than 1 indicating that a low tPSA is correctly associated with not having the disease. However only one meets the accepted level of less than 0.1.

**Figure 6:**
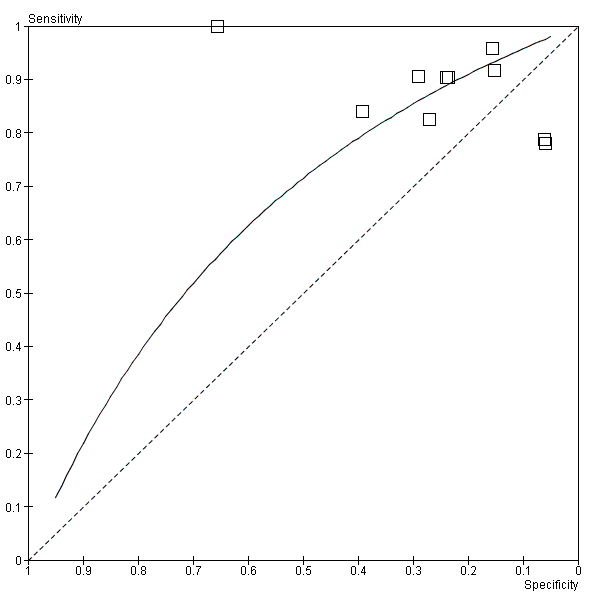
**SROC Plot of tPSA testing**.

**Figure 7:**
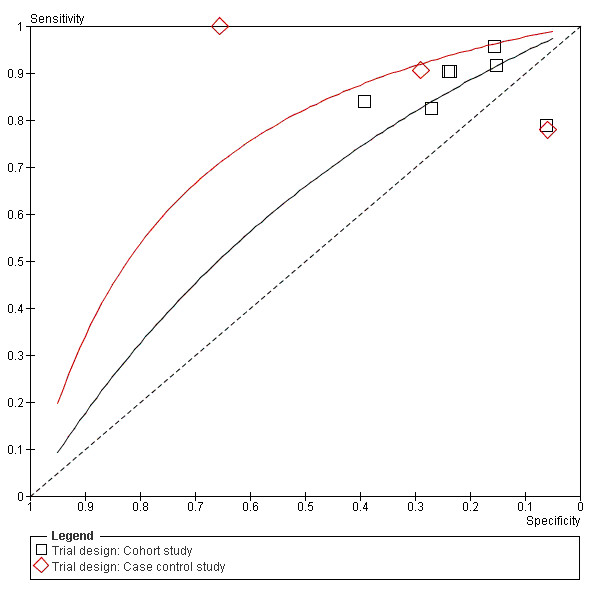
**Summary ROC Plot of PSA testing taking account of trial design**.

The SROC curve [Figure 6] lies to the left of the diagonal signifying that the tPSA test has value. The SROC analysis was further developed by placing the studies into subgroups based up trial design. [Figure 7].

### Subgroup analysis

**Figure 8:**
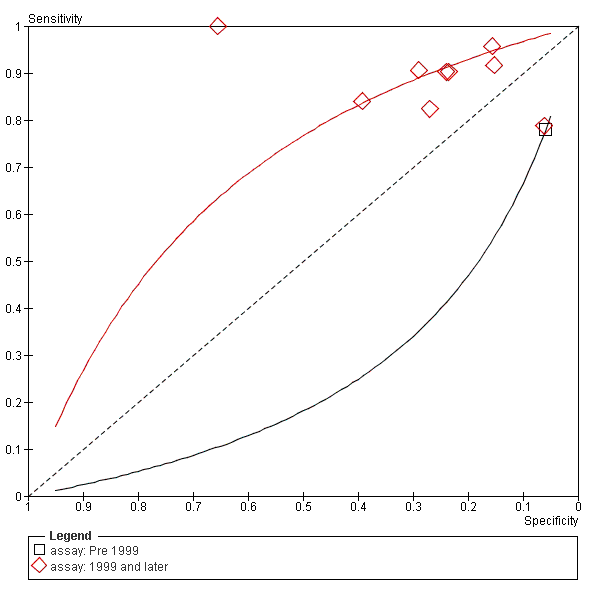
**SROC curves comparing the study using the pre 1999 PSA assay and the studies using assays from 1999 and onwards**.

To explore the heterogeneity, subgroups of cohort and case control, and pre-1999 and post-1999 were created. Summary receiver operating characteristic (SROC) curves were plotted for each subgroup. Figure 7 shows greater test accuracy in the case control subgroup, figure 8 shows test accuracy was greater post-1999, most likely due to the WHO guidelines instigated that year. Outlying studies; Espana 1998, Wymenga 2000 and Unal 2000, can be accounted for by either case-control design or pre-1999 assays.

## Discussion

Overall tPSA seems to have significance as a diagnostic tool. DORs ranged from 0.221 to 4.16. All but two studies gave DORs greater than 1. The SROC curve [Figure 6] lies to the left of the diagonal signifying that the PSA test has value. Adequate levels of sensitivity appear to be achieved at the expense of poor specificity, with consequently relatively high numbers of false positive results.

Espana 1998 and Wymenga 2000 had DORs below 1. This suggests that the PSA of more than 4 ng/ml is inversely associated with prostate cancer. Potential sources of bias for Espana 1998 are its age and poor explanation of withdrawn participants. Its small size makes it especially vulnerable to these factors. Wymenga 2000 was a cohort study which did not exclude borderline cases of raised PSA. This gives the appearance of poorer test accuracy, compared to a case-control study.

Unal 2000's isolated position in the top left on the SROC curve produces the most significant results supporting the use of tPSA as a diagnostic test. Its false negative rate of 0 is likely to be due to the small size of the study and its case control design. Despite its outlying results its high methodological quality warrants its inclusion.

In contrast, Aragona 2005 is the largest included study and has the narrowest CI. Its sensitivity and specificity lies within the main cluster of results close to the SROC curve, adding weight to our findings.

### Strengths and weaknesses of the review

A large number of abstracts were reviewed. With regards to study design, suitable publications may have been omitted due to the sole use of electronic searches, reviewer error or limited search terms. Further detail could be added to the searches, including the use of limited text terms. Publication bias may occur but there is no consensus on its importance [14] or how to assess the impact on this on systematic reviews of diagnostic test accuracy [15]. As the current use of tPSA in clinical practice is debated, it is unclear whether publication bias would exclude papers showing a low test accuracy or high test accuracy.

The populations of our studies were limited to men attending urology clinics because of referral due to clinical evidence in primary or secondary care, such as an abnormal DRE or raised PSA. This means that our results cannot be applied to the screening population. In this setting there would be a lower prevalence of prostate cancer so it is likely that PSA would have less accuracy as a diagnostic test since the specificity has been shown to be low. Also, the populations studied were European men. A more detailed breakdown of the race of the study populations would have provided us with more information on sources of heterogeneity. Overall the results can be applied to symptomatic European men in the primary and secondary healthcare setting.

The subgroup analyses can show valuable results, however there are some limitations. Firstly, there is overlap between case control and cohort studies. It was sometimes difficult to place the studies into these categories. Secondly, for our pre- and post- 1999 analysis there was only 1 study in the pre-1999 group.

Pre-1999 many assays were widely used for the detection of total PSA, for example Tandem-E, Tandem-R, Immulite 2000, ADIVA Centaur and Roche 2. There has been significant difference in the results using the various assays [16-18]. Also as mentioned earlier, in 1999 the World Health Organisation established a reference standard for total PSA measurement[5]. Since then, differences have decreased between the results of different assay methods [6].

Ultimately, the lack of large studies on Europeans which were suitable for our analysis was the main limitation of this review.

### Applicability of findings to clinical practice and policy

PSA testing is clearly a vital part of the diagnostic pathway. We have previously discussed the limitations of the study populations. However, we can apply our results to patients who are referred for a biopsy. This is useful for general practitioners and urologists to reassure patients with a raised PSA.

We have found that the PSA test had a sensitivity ranging from 0.78 to 1.00, which means it potentially fails to diagnose over 20% of prostate cancers. This is important to consider in patients with continuing symptoms or an isolated, abnormal DRE. Good quality counselling and information needs to be given to patients to ensure they present again if symptoms persist or worsen. The DRE needs to remain a key part in the diagnostic pathway.

PSA is known to have low specificity, however our results show an extremely low range of 0.06 to 0.66. All but Unal 2000 showed a specificity of less than 0.40. This is in contrast to a moderate specificity as stated by NICE in the most recent guidelines on referral practice for suspected cancer in adults and children. Such a low specificity means that in practice many patients are undergoing the invasive procedure of biopsy who do not in fact have prostate cancer. However there is currently no alternative that has been recommended by NICE for use in clinical practice. It might be interesting to sub-analyse the data according to the patients' presenting symptoms, as this would be useful in the assessment of PSA as both a diagnostic test and a screening tool.

## Conclusion

### Implications for practice

PSA testing still has a role to play in the diagnostic pathway and is relatively non-invasive and inexpensive [19]. However it has a low specificity. Other tests, which could improve this, are currently being researched but have not been implemented into clinical practice. We recommend that PSA testing continues to be used in clinical practice as one of the several indicators for biopsy, but it is important that clinicians understand the limitations of the test. It would be interesting to assess the role of a PSA result in GPs' decision making, for example the impact of the high false positive and significant false negative rates.

### Implications for research

As our study was unable to assess specificity and sensitivity in the screening setting it would be useful for a review to examine this. Screening is especially relevant as programmes emerge. We would recommend that for further research done into PSA, the STARD statements be implemented to ensure standardisation and transparency. We recommend that further research includes a sub-analysis according to patients' symptoms.

## Authors' contributions

All authors were involved in writing of the protocol, reviewing of papers and selection for inclusion, writing of the paper itself, analysis, statistical interpretation and revisions. In addition PH constructed figures and performed the statistical analysis. DE extracted the data from the included studies and constructed the results section. AB and AI also took part in writing the results section and also wrote the introduction and objectives sections. BC performed the medline and embase searches and wrote the corresponding part of the methods section. MW was the main collator of papers and was also involved in data extraction and statistical analysis.

## Pre-publication history

The pre-publication history for this paper can be accessed here:



## Supplementary Material

Additional file 1 **QUADAS criteria**. Criteria used to assess papers for methodological quality. Click here for file

Additional file 2 **Reasons for exclusion of studies**. A list of short listed studies that was not included with reasons for exclusion. Click here for file

Additional file 3 **Tables of study features with individual trial methodological quality tables**. Detailed assessment of study characteristics and methodological quality. Click here for file
