# Supplementary material for: A systematic review of the diagnostic accuracy of prostate specific antigen
Source: BMC Urol. 2009 Sep 10;9:14. doi: 10.1186/1471-2490-9-14 (PMC2753579; doi:10.1186/1471-2490-9-14)
Supplement: Additional file 1 — QUADAS criteria. Criteria used to assess papers for methodological quality. [file 1471-2490-9-14-S1.doc]

Additional file 1

QUADAS criteria:

1. How representative the participants were of the population of European males. We also recorded details of how participants were selected for the studies, as this affects which type of patients we can apply the results to. For example, patients referred for biopsy.

2. Avoidance of a partial verification of the target condition - whether the reference test (histology) was only performed on those patients with a positive index test (tPSA ≥4ng/mL)

3. Acceptability of the reference standard used to give a final diagnosis - the accuracy of the histology in the diagnosis of prostate cancer. Histological examination of a biopsy or a resected specimen was deemed acceptable.

4. Blinding of results of index test when reference test result was interpreted. Histological examination is relatively objective, so it is unlikely that lack of blinding will be a significant cause of bias. However it is possible that a borderline specimen will be interpreted differently if the researcher knows the tPSA is above or below 4ng/ml.

5. Blinding of results of reference test when the index test was interpreted. The tPSA level is an objective numerical test, so it is very unlikely that lack of blinding will lead to bias.

6. Availability of relevant clinical data when all test results were interpreted. The same clinical data should be available when interpreting test results as are available in normal clinical practice.

7. Reporting of uninterpretable results - Uninterpretable results are more likely than others to be borderline diagnoses and so would have a greater effect on test accuracy if included.

8. Reporting and explanation of withdrawals - if patients were lost to follow-up and reasons for this.

Further work to be done……Additional statistics (hierarchical SROC and bivariate methods) can be calculated by exporting data from RevMan into Statistical Analysis System (SAS) or equivalent statistical packages, but such additional analysis was not employed.
