# Supplementary material for: A systematic review of the diagnostic accuracy of prostate specific antigen
Source: BMC Urol. 2009 Sep 10;9:14. doi: 10.1186/1471-2490-9-14 (PMC2753579; doi:10.1186/1471-2490-9-14)
Supplement: Additional file 2 — Reasons for exclusion of studies. A list of short listed studies that was not included with reasons for exclusion. [file 1471-2490-9-14-S2.doc]

**Additional file 2**

**Reasons for exclusion of studies**

| Study name | Reason for exclusion |
| --- | --- |
| Andriole 2005 | Not all patients received biopsies |
| Aus 2001 | Not all patients underwent biopsy |
| Aus 2005 | Data collected before 1998 |
| Auvinen 2004 | Limited PSA range below 4ng/ml |
| Barette 2001 | Not all patients underwent biopsy |
| Basso 2000 | Relevant data was not extractable |
| Ciatto 2001 | Some data collected before 1998 |
| Djavan 2002 | Relevant data was not extractable |
| Emiliozzi 2004 | Only patients with a PSA over 4 ng/ml were included |
| Filella 2001 | Not all patients underwent biopsy |
| Filella 2004 | Relevant data was not extractable |
| Filella 2007 | Relevant data was not extractable |
| Finne 2002 | Data collected before 1998 |
| Galic 2003 | Not all patients underwent biopsy |
| Gustafsson 1998 | Relevant data was not extractable |
| Hakama 2001 | Data was collected before 1998 |
| Horninger 2000 | Data was collected before 1998 |
| Horninger 2001 | Only a limited range of PSA values were used |
| Irani 2005 | Relevant data was not extractable |
| Jung 1998 | Data collected before 1998 |
| Koliakos 2000 | Data collected before 1998 |
| Lein 2001 | Relevant data was not extractable |
| Lein 2001 (2) | Not all patients underwent biopsy |
| Lein 2005 | Incomplete Data |
| Lin 1998 | Data collected before 1998 |
| Luboldt 2001 | Not all patients underwent biopsy |
| Maattanen 2001 | Data collected before 1998 |
| Maattanen 2007 | Data collected before 1998 (96) |
| Makinen 2001 | Not all patients underwent biopsy |
| Martin 1999 | Data collected before 1998 |
| Martínez-Piñeiro 2004 | Data collection started before 1998 |
| Mitchell 2001 | Relevant data was not extractable |
| Morote 2002 | A limited range of PSA values was used |
| Raaijmakers 2003 | Patients with PSA of less than 3 ng/ml did not undergo biopsy |
| Ravery 1999 | Relevant data was not extractable |
| Romppanen 2002 | Relevant data was not extractable |
| Rowe 2005 | Not all patients received a biopsy |
| Shroeder 1998 | Data collected before 1998 |
| Stephan 2005 | Patients were enrolled before 1998 |
| Stephan 2007 | Only used patients with PSA of between 0.5 and 10ng/ml |
| Tello 2001 | Relevant data was not extractable |
| Vis 2001 | Only patients with PSA of less than 4 ng/ml were included in the study |
| Wymenga 2001 | Limited range of PSA values were used |
| Zhang 2000 | Relevant data was not extractable |
