# Supplementary material for: A systematic review of the diagnostic accuracy of prostate specific antigen
Source: BMC Urol. 2009 Sep 10;9:14. doi: 10.1186/1471-2490-9-14 (PMC2753579; doi:10.1186/1471-2490-9-14)
Supplement: Additional file 3 — Tables of study features with individual trial methodological quality tables. Detailed assessment of study characteristics and methodological quality. [file 1471-2490-9-14-S3.doc]

### Additional file 3

### Tables of study features with individual trial methodological quality tables

### Aragona 2005

| Clinical features and settings | Not screening. Patient cohort created from previous urology outpatient referrals. These were recalled for PSA testing and DRE, followed by biopsy if either was abnormal. In the case of PSA this included values above 10ng/ml. Also those below 2.5ng/ml, 2.6ng/ml - 4ng/ml or a PSA between 4.1-10ng/ml. These are combined with free:total PSA ratio of less than or equal to 15%, 20% and 25% respectively. |
| --- | --- |
| Participants | 3171 |
| Study design | Cohort |
| Target condition and reference standard(s) | Prostate Ca by ultrasound guided transrectal or transperineal (needle count unspecified) prostate biopsy |
| Index and comparator tests | Roche 2 immunoassay |
| Follow-up | N/A |
| Notes |  |

#### Assessment of methodological quality table

| **Item** | **Judgement** | **Description** |
| --- | --- | --- |
| Representative spectrum? | Yes |  |
| Acceptable reference standard? | Yes |  |
| Partial verification avoided? | Yes |  |
| Reference standard results blinded? | Unclear |  |
| Index test results blinded? | Unclear |  |
| Relevant clinical information? | Yes |  |
| Uninterpretable results reported? | No | 8 patients with unexplained findings |
| Withdrawals explained? | Yes | 95 patients refused biopsy |

### Beneduce 2007

| Clinical features and settings | Not screening. Patients taken from those with symptoms suggestive of prostate cancer referred for biopsy. 15 healthy volunteers and 5 prostate cancer patients added to the cohort. |
| --- | --- |
| Participants | 101 |
| Study design | Cohort |
| Target condition and reference standard(s) | 10 or 12 core TRUS guided biopsy dependent on prostate size, less than or greater than 35cm (cubic) respectively |
| Index and comparator tests | chemoluminescent immunometric assay |
| Follow-up | after one year no BPH patients developed Prostate Ca |
| Notes |  |

#### Assessment of methodological quality table

| **Item** | **Judgment** | **Description** |
| --- | --- | --- |
| Representative spectrum? | Yes | All patients received biopsies, sample reflects this |
| Acceptable reference standard? | Yes |  |
| Partial verification avoided? | Yes |  |
| Reference standard results blinded? | Yes |  |
| Index test results blinded? | Unclear |  |
| Relevant clinical information? | Yes |  |
| Uninterpretable results reported? | Unclear | none declared |
| Withdrawals explained? | Unclear | No withdrawals |

### Ciatto 2004

| Clinical features and settings | Not screening. Patient group drawn from consecutive biopsies recommended due to abnormal DRE, TRUS or raised PSA. |
| --- | --- |
| Participants | 410 outpatients |
| Study design | Cohort |
| Target condition and reference standard(s) | Prostate Ca by TRUS guided transperineal either sextant random biopsy or directed biopsy |
| Index and comparator tests | Tandem-R PSA |
| Follow-up | N/A |
| Notes |  |

#### Assessment of methodological quality table

| **Item** | **Judgment** | **Description** |
| --- | --- | --- |
| Representative spectrum? | Yes | All patients had biopsies hence the spectrum of patients involved is reflective of this. |
| Acceptable reference standard? | Yes |  |
| Partial verification avoided? | Yes |  |
| Reference standard results blinded? | Unclear |  |
| Index test results blinded? | Unclear |  |
| Relevant clinical information? | Yes |  |
| Uninterpretable results reported? | Unclear | None reported |
| Withdrawals explained? | Unclear | No withdrawals |

### Espana 1998

| Clinical features and settings | Non screening. Patient group composed of those attending urology department for biopsy |
| --- | --- |
| Participants | 170 |
| Study design | Case control |
| Target condition and reference standard(s) | Prostate Ca by TRUS guided sextant needle biopsy |
| Index and comparator tests | Unknown |
| Follow-up | N/A |
| Notes |  |

#### Assessment of methodological quality table

| **Item** | **Judgment** | **Description** |
| --- | --- | --- |
| Representative spectrum? | Yes | All patients fulfilled criteria for biopsy |
| Acceptable reference standard? | Yes |  |
| Partial verification avoided? | Yes |  |
| Reference standard results blinded? | Unclear | Probably had earlier PSA results available at time of biopsy, but not the ones used in the study |
| Index test results blinded? | No |  |
| Relevant clinical information? | Yes |  |
| Uninterpretable results reported? | Unclear |  |
| Withdrawals explained? | No | Initially 250 patients had biopsy taken. 191 were diagnosed with BPH. Only the first 111 of these patients were included in the PSA testing |

### Fischer 2005

| Clinical features and settings | Not screening. Patient group taken from in- and outpatients at a urology clinic. |
| --- | --- |
| Participants | 178, 74 untreated Prostate cancer patients, the remainder had other non malignant prostatic illness. |
| Study design | Cohort |
| Target condition and reference standard(s) | Prostate Ca by histological verification from a sample by prostate puncture cylinder or resection. |
| Index and comparator tests | Immulite |
| Follow-up | N/A |
| Notes |  |

#### Assessment of methodological quality table

| **Item** | **Judgment** | **Description** |
| --- | --- | --- |
| Representative spectrum? | Yes | Comparison of Prostate Ca group with a BPH control group |
| Acceptable reference standard? | Yes | Histological diagnosis |
| Partial verification avoided? | Yes |  |
| Reference standard results blinded? | Unclear |  |
| Index test results blinded? | Unclear |  |
| Relevant clinical information? | Yes |  |
| Uninterpretable results reported? | Unclear | None disclosed |
| Withdrawals explained? | Unclear | No withdrawals |

### Hofer 2000

| Clinical features and settings | Not screening. Patients with histological diagnosis by TRUS guided biopsy, TURP or suprapubic adenomectomy |
| --- | --- |
| Participants | 184 |
| Study design | Case control |
| Target condition and reference standard(s) | Prostate Ca by histological examination of tissue from either biopsy or resection |
| Index and comparator tests | Tandem-e PSA |
| Follow-up | N/A |
| Notes |  |

#### Assessment of methodological quality table

| **Item** | **Judgment** | **Description** |
| --- | --- | --- |
| Representative spectrum? | Yes | Patient group selected by prior diagnosis of Prostate Ca |
| Acceptable reference standard? | Yes |  |
| Partial verification avoided? | Yes |  |
| Reference standard results blinded? | Unclear |  |
| Index test results blinded? | Unclear |  |
| Relevant clinical information? | Yes |  |
| Uninterpretable results reported? | Unclear | None declared |
| Withdrawals explained? | Unclear | No withdrawals |

### McArdle 2004

| Clinical features and settings | Not screening. The patient group came from those undergoing consecutive biopsies following positive PSA test or DRE |
| --- | --- |
| Participants | 171 |
| Study design | Cohort study |
| Target condition and reference standard(s) | Prostate Ca by sextant (or more) biopsy |
| Index and comparator tests | PSA Bayer ADVIA centaur assay |
| Follow-up | N/A |
| Notes |  |

#### Assessment of methodological quality table

| **Item** | **Judgment** | **Description** |
| --- | --- | --- |
| Representative spectrum? | Yes | Patients only incorporated if they have a raised PSA or abnormal DRE. |
| Acceptable reference standard? | Yes |  |
| Partial verification avoided? | Yes |  |
| Reference standard results blinded? | Unclear |  |
| Index test results blinded? | Unclear |  |
| Relevant clinical information? | Yes |  |
| Uninterpretable results reported? | Unclear |  |
| Withdrawals explained? | Unclear |  |

### Ryden 2007

| Clinical features and settings | Not screening. The patient group came from those undergoing consecutive biopsy following either: abnormal DRE, PSA ratio less than 0.18 or PSA above 4. |
| --- | --- |
| Participants | 361, 326 had PSA over 4, 24 had PSA ratio under 0.18 and 11 had abnormal DRE results. |
| Study design | Cohort study |
| Target condition and reference standard(s) | Prostate Ca by TRUS guided biopsy from end firing ultrasound probe using "biopince" biopsy |
| Index and comparator tests | PSA, unspecified assay |
| Follow-up | N/A |
| Notes |  |

#### Assessment of methodological quality table

| **Item** | **Judgment** | **Description** |
| --- | --- | --- |
| Representative spectrum? | Yes | Consecutive patients undergoing biopsy |
| Acceptable reference standard? | Yes |  |
| Partial verification avoided? | Yes |  |
| Reference standard results blinded? | Unclear |  |
| Index test results blinded? | Unclear |  |
| Relevant clinical information? | Yes |  |
| Uninterpretable results reported? | Unclear | None |
| Withdrawals explained? | Unclear | No withdrawals |

### Unal 2000

| Clinical features and settings | Not screening. Patient group taken from those with diagnosis and scheduled to undergo radical prostatectomy or transurethral microwave therapy. |
| --- | --- |
| Participants | 59 participants, 30 with localised prostate cancer, 29 with BPH |
| Study design | Case control |
| Target condition and reference standard(s) | Prostate cancer by biopsy (not specified further) |
| Index and comparator tests | Tandem PSA |
| Follow-up | N/A |
| Notes |  |

#### Assessment of methodological quality table

| **Item** | **Judgment** | **Description** |
| --- | --- | --- |
| Representative spectrum? | Yes | All controls have BPH and are scheduled for transurethral microwave therapy |
| Acceptable reference standard? | Yes |  |
| Partial verification avoided? | Yes |  |
| Reference standard results blinded? | Unclear |  |
| Index test results blinded? | No |  |
| Relevant clinical information? | Unclear | Biopsy results available at time of testing |
| Uninterpretable results reported? | Unclear |  |
| Withdrawals explained? | Unclear | No withdrawals |

### Wymenga 2000

| Clinical features and settings | Non screening. Patient group from urology outpatient referrals due to: micturation disorders, abnormal DRE, findings suggestive of prostate Ca or raised PSA. |
| --- | --- |
| Participants | 716 |
| Study design | Cohort |
| Target condition and reference standard(s) | Prostate Cancer by TRUS-guided sextant biopsy or histological examination of surgically resected specimens |
| Index and comparator tests | IMMULITE immnoreactive total PSA assay |
| Follow-up | N/A |
| Notes |  |

#### Assessment of methodological quality table

| **Item** | **Judgment** | **Description** |
| --- | --- | --- |
| Representative spectrum? | Yes | Only patients with either elevated PSA, abnormal DRE or abnormal TRUS included |
| Acceptable reference standard? | Yes |  |
| Partial verification avoided? | Yes |  |
| Reference standard results blinded? | Unclear |  |
| Index test results blinded? | Yes |  |
| Relevant clinical information? | Yes |  |
| Uninterpretable results reported? | Unclear |  |
| Withdrawals explained? | Unclear | No withdrawals |
